# Multi-centre, randomised, open-label, blinded endpoint assessed, trial of corticosteroids plus intravenous immunoglobulin (IVIG) and aspirin, versus IVIG and aspirin for prevention of coronary artery aneurysms (CAA) in Kawasaki disease (KD): the KD CAA prevention (KD-CAAP) trial protocol

**DOI:** 10.1186/s13063-022-07051-9

**Published:** 2023-01-26

**Authors:** Despina Eleftheriou, Yolanda Collaco Moraes, Cara Purvis, Molly Pursell, Marta Merida Morillas, Robin Kahn, Maria Mossberg, Filip Kucera, Robert Tulloh, Joseph F. Standing, Veronica Swallow, Rachael McCormack, Jethro Herberg, Michael Levin, Mandy Wan, Nigel Klein, Roisin Connon, Ann Sarah Walker, Paul Brogan

**Affiliations:** 1 UCL Great Ormond Street Institute of Child Health, 30 Guilford Street, London, WC1N 1EH UK; 2 Medical Research Council (MRC) Clinical Trials Unit (CTU) at University College London (UCL), London, UK; 3 Department of Paediatrics, Lund University, Clinical Sciences, Lund, Sweden; 4 Great Ormond Street Hospital, London, UK; 5 Bristol Heart Institute, Bristol, UK; 6 Sheffield Hallam University, Sheffield, UK; 7 Societi Foundation CIO, The UK Foundation for Kawasaki Disease, Newark, UK; 8 Section of Paediatric Infectious Diseases, Imperial College London, London, UK; 9 Pharmacy Department, Evelina London Children’s Hospital, Guy’s and St Thomas’ NHS Foundation Trust, London, UK; 10 Institute of Pharmaceutical Science, King’s College London, London, UK

**Keywords:** Kawasaki disease, Corticosteroids, Coronary artery aneurysms

## Abstract

**Background:**

Kawasaki disease (KD) is an acute self-limiting inflammatory vasculitis affecting predominantly medium-sized arteries, particularly the coronary arteries. A number of recent studies conducted in different European countries have demonstrated alarmingly high coronary complications despite treatment with intravenous immunoglobulin (IVIG). These high complication rates now emphasize the need for an urgent reappraisal of IVIG as the sole primary therapeutic agent for KD. The Kawasaki disease CAA prevention (KD-CAAP) trial will test the hypothesis that immediate adjunctive corticosteroid treatment to standard of care IVIG and aspirin will reduce coronary artery aneurysm (CAA) rates in unselected KD patients across Europe.

**Methods:**

KD-CAAP is a multicentre, randomised, controlled, open-label, blinded endpoint assessed trial that will be conducted across Europe supported by the conect4children pan-European clinical trials network. Patients with KD who satisfy the eligibility criteria will be randomised (1:1) to receive either oral prednisolone 2 mg/kg/day plus standard of care therapy IVIG (2 g/kg) and aspirin (40 mg/kg/day); or IVIG and aspirin alone. Further management is dictated by temperature and C-reactive protein (CRP) responses. Co-primary outcomes are as follows: (i) any CAA within the 3 months of trial follow-up; (ii) average estimate of maximum coronary *Z*-score at weeks 1, 2 and 6 adjusting for rescue treatment. Additional outcomes will be assessed including cost effectiveness, quality of life, corticosteroid toxicity and other safety outcomes.

**Discussion:**

Several recent studies have indicated that coronary complications associated with KD across Europe are much higher than early trials of IVIG had initially suggested. KD-CAAP directly addresses this issue by exploring the therapeutic benefit of adjunctive corticosteroids in unselected KD cases. If we find that corticosteroids prevent CAA and are safe, this is a cheap and widely available intervention that could be implemented immediately for the benefit of children.

**Trial registration:**

ISRCTN71987471- March 31, 2020; Eudract 2019–004433-17.

## Administrative information

Note: The numbers in curly brackets in this protocol refer to SPIRIT checklist item numbers. The order of the items has been modified to group similar items (see).

**Table:** 

**Title {1}**	**Multi-centre, randomised, open-label, blinded endpoint assessed, trial of corticosteroids plus intravenous immunoglobulin (IVIG) and aspirin, versus IVIG and aspirin alone for prevention of coronary artery aneurysms in Kawasaki diseaseShort acronym: KD-CAAP (Kawasaki Disease Coronary Artery Aneurysm Prevention trial)**
**Trial registration {2a and 2b}.**	**ISRCTN71987471** **Eudract: 2019–004433-17**
**Protocol version {3}**	**Protocol version 5.0**
**Funding {4}**	Funding is provided by the Innovative Medicines Initiative 2 Joint Undertaking (JU), under grant agreement No 777389 that supports the conect4children (c4c) research consortium
**Author details {5a}**	^1^Despina Eleftheriou, ^2^Yolanda Collaco Moraes, ^2^Cara Purvis, ^2^Molly Pursell, ^2^Marta Merida Morillas ^2^Nigel Klein, ^3^Robin Kahn, ^3^Maria Mossberg, ^4^Filip Kucera, ^5^Robert Tulloh, ^1^Joseph F Standing, ^6^Veronica Swallow, ^7^Rachael McCormack, ^8^Jethro Herberg, ^8^Mike Levin, ^9^Mandy Wan, ^2^Roisin Connon, ^2^ Ann Sarah Walker, ^1^Paul Brogan 1. UCL Great Ormond Street Institute of Child Health, London, UK; 2. Medical Research Council (MRC) Clinical Trials Unit (CTU) at University College London (UCL), London, UK; 3 Lund University, Department of Paediatrics, Clinical Sciences, Lund, Sweden; 4 Great Ormond Street Hospital, London, UK; 5 Bristol Heart Institute, Bristol, UK; 6 Sheffield Hallam University, UK; 7 Societi Foundation, the UK Foundation for Kawasaki Disease, Newark, UK; 8. Section of paediatric infectious diseases, Imperial College London, UK; 9. Pharmacy Department, Evelina London Children's Hospital, Guy's and St Thomas' NHS Foundation Trust, London, UK; 10. Institute of Pharmaceutical Science, King's College London, London, UK.
**Name and contact information for the trial sponsor {5b}**	University College London Gower Street London WC1E 6BT Contact: mrcctu.kdcaap@ucl.ac.uk
**Role of sponsor {5c}**	UCL have delegated sponsor responsibilities to the MRC CTU at UCL who have provided input into the trial design and the drafting of this publication. The MRC CTU at UCL together with the Trial Management Group and the Chief Investigators will have oversight of the data collection, analysis and interpretation and the management for the trial. The KD-CAAP Trial Steering Committee is custodian for the data and specimens generated from the KD-CAAP trial; KD CAAP trial data are not the property of individual participating investigators or health care facilities where the data were generated.

## Introduction

### Background and rationale {6a}

#### Kawasaki disease (KD)

KD is an acute self-limiting inflammatory vasculitis affecting predominantly medium-sized arteries, particularly the coronary arteries causing coronary artery aneurysms (CAA) [1–4]. KD is currently the commonest cause of acquired heart disease in children in high-income countries [1–4]. KD causes CAA in 15–25% of untreated patients whilst 2–3% of untreated cases die as a result of coronary vasculitis [1–4]. Coronary artery vasculitis can cause acute myocardial events in the early stages of the disease leading to myocardial infarction or even death [1–4]. Late morbidity can also arise from late KD vasculopathy, a process involving remodelling following the acute inflammatory event, distinct from atherosclerosis, but ultimately leading to coronary vascular insufficiency and late cardiac events [1–4]. Notably, as more children with KD survive into adulthood, the disease remains an important cause of long-term cardiac disease in adulthood and requires rigorous follow-up, particularly for those with persisting/giant CAA, to reduce risk of myocardial ischaemia and infarction [1–4].

The disease has a world-wide distribution with higher risk in males (male: female case ratio of 1.5: 1), seasonality and occasional epidemics [1–4]. KD is more prevalent in Japanese children (308/100,000 under the age of 5 years). An increased incidence of KD is also observed in Japanese and other Asian children resident in North America and Europe, suggesting a genetic contribution [1–8]. In the UK, a recent direct British Paediatric Surveillance Unit epidemiological survey (2013–2015) showed that the incidence of KD in the UK and Ireland was 4.55/100,000 children under 5 years, which represents a slight increase since the last survey in 1990 [4, 9]. Whilst the majority of cases were Caucasian, KD in the UK is over-represented in Chinese or Japanese Asians and Black Africans [4, 9].

#### Current treatment of KD and reported rates of coronary complications

Randomised controlled trials and meta-analyses have unequivocally demonstrated that early recognition and treatment of KD with intravenous immunoglobulin (IVIG) and aspirin reduces the occurrence of CAA [1–3, 10–12]. Therefore, IVIG and aspirin should be started as soon as a patient is diagnosed with complete or incomplete KD [1, 2]. Two grams per kilogram of IVIG is the recommended dose, usually given as a single infusion (typically over 12 h), in view of greater therapeutic effect in preventing CAA when compared to a lower, divided dose regimen [13]. Close monitoring of patients is critical, considering temperature, acute phase reactants (particularly C-reactive protein; CRP), clinical symptoms and other signs of systemic inflammation. All patients should also initially receive aspirin at a dose of 30–50 mg/kg/day, in 3–4 divided doses [1–3, 14]. Aspirin should be reduced to an antiplatelet dose of 3–5 mg/kg/day, but only after the fever has settled for 48 h, clinical features are improving, and CRP levels are falling in line with CRP half-life (approximately 18 h in the absence of ongoing hepatic production) [1–3, 14].

Early recognition and treatment of KD with aspirin (30–50 mg/kg/day, in 3–4 divided doses) and intravenous immunoglobulin (IVIG; 2 g/kg) was previously thought to reduce the risk of occurrence of CAA from approximately 20% in untreated patients to 4% [1–3]. IVIG resistance occurs in up to 20–40% of cases, however, and this IVIG resistance is associated with increased risk of developing CAA [1–4]. Notably however, several recent studies conducted in Europe (UK, Sweden and Germany), Russia and North America have found alarmingly high rates of coronary complications despite IVIG [4, 8, 15–17]. A recent UK survey (2013–2015) suggested that 19% of children with KD developed CAA despite IVIG; and, even more worryingly, 39% of those under 1 year old developed CAA [4]. Similarly, in Germany overall CAA rates of 22% (42% in younger children) have been reported despite treatment with IVIG [17]. In Skane, Sweden, the overall rate of CAA in a recent survey was reported as 16% despite IVIG, with 45% under the age of 1 year developing CAA [8].

The reasons for these alarmingly high rates of CAA are currently unclear. Late diagnosis is of concern (particularly for those with incomplete KD) and delayed treatment undoubtedly plays a role, since in the latest UK survey, time to IVIG treatment was found to be delayed in those with CAA compared with those without CAA. Additionally, Caucasians may not respond as well to IVIG as non-Caucasians, perhaps due to an as yet unidentified pharmacogenomic differences, such as the hypothesis with respects to Fc gamma-receptor polymorphisms that may influence IVIG-responsiveness in different populations. Whatever the reason(s), these very high CAA complication rates now emphasise the need for an urgent reappraisal of IVIG as the primary therapeutic agent for KD.

#### Corticosteroids for the treatment of KD: efficacy and safety data

Corticosteroids are an effective treatment for virtually all forms of vasculitis, but they have not been widely adopted as first-line treatment in unselected KD cases [1–3]. This is largely due to conflicting efficacy data from previous clinical trials in non-European patients using very different corticosteroid dosing regimens in patients with differing risk profiles for CAA. This is illustrated when the American Paediatric Heart Network trial and the Japanese RAISE trial (two of the largest RCTs examining this issue) are considered in more detail [18, 19]. Both these trials investigated the use of corticosteroids in addition to standard IVIG and aspirin [18, 19]. The American trial evaluated the use of intravenous methylprednisolone (30 mg/kg) given as a single dose combined with IVIG in unselected patients with KD [19]. In contrast, the RAISE trial evaluated different dose (2 mg/kg) intravenous methylprednisolone given for 5 days; if fever settled, this was then converted to oral prednisolone which was subsequently tapered over 15 days after the C-reactive protein (CRP) normalised [18]. Moreover, patients were included in RAISE only if they were at high risk of IVIG resistance, based on a risk score (Kobayashi score ≥ 5). Perhaps unsurprisingly then, these two studies produced different results, with corticosteroids conferring significant benefit in the Japanese RAISE trial, but a lack of overall benefit in the American trial, possibly because this latter trial did not use enough corticosteroid. Importantly, a large ‘post-RAISE’ observational study of 724 high-risk Japanese patients receiving corticosteroid treatment in addition to IVIG and aspirin showed that primary IVIG plus prednisolone therapy had an effect similar to that seen in the RAISE trial, and significantly reduced the incidence of CAA with minimal adverse events [20]. This observational analysis provides additional ‘real-world’ support for the use of corticosteroids in high-risk KD cases in Japan.

Notably, meta-analysis of data from several published studies of corticosteroid therapy in KD also provides evidence supporting the use of corticosteroids as primary adjunctive treatment for patients with severe KD [21]. Meta-analysis of 16 comparative studies (mostly observational, few randomised) involving 2746 KD patients demonstrated that early addition of corticosteroids to conventional IVIG therapy was associated with reduced risk of CAA compared with IVIG therapy alone (odds ratio 0.424; 95%CI, 0.270–0.665) [21]. This benefit was only observed when corticosteroids were used as primary therapy rather than rescue therapy for IVIG resistance, and was greatest for Japanese patients who were determined at baseline to have high risk for IVIG resistance. Meta-regression analyses also demonstrated that corticosteroids were more effective when started earlier in the disease course. Overall, this meta-analysis provided evidence that corticosteroids combined with IVIG as initial treatment reduces overall risk of CAA in severe KD, but did not resolve the ongoing debate about which KD patients should receive this, probably explaining why less than 5% of patients in the UK currently receive corticosteroids as primary adjunctive treatment [21]. A high-risk patient is regarded as one where the risk of CAA is 20–30% despite IVIG treatment, and in Japan is identified as those patients with a Kobayashi score ≥ 5 [22]. For UK and European patients, however, the Kobayashi risk score had poor sensitivity to identify patients at higher risk of CAA [23]. In line with this evidence, recent European SHARE guidelines for KD recommend adjunctive corticosteroids for high-risk patients, but acknowledge that identifying such patients in Caucasian populations is difficult and that clinical scores to define high risk patients developed for Japanese patients perform sub-optimally in Caucasians [2]. However, given the high CAA rates emerging from several countries, and the lack of risk assessment tools to accurately identify such cases, it is reasonable now to argue that all European KD patients are at significant risk of CAA despite IVIG (19–45%) and could potentially benefit from primary treatment with corticosteroids.

Several previous studies have also indicated that short courses of corticosteroids were safe in KD. Specifically, in the American Paediatric Heart Network trial that evaluated the use of intravenous methylprednisolone (30 mg/kg) given as a single dose combined with IVIG in unselected patients with KD (experimental group), compared to controls who received placebo plus IVIG, there was no evidence of differences in adverse events reported in both trial groups: 37/101 (36%) in the experimental group compared to 24/97 (25%) in the control group, *p* = 0.18 [19]. The majority of these adverse events were judged to be related to IVIG use and were not related to corticosteroids. The few adverse events clearly attributed to corticosteroid use, observed in only 5/101 patients, included hypotension, and one episode of hypokalaemia; all were quickly resolved with no intervention. Similar favourable safety data have been reported from studies of corticosteroids in Japan. The Japanese RAISE trial evaluated different dose (2 mg/kg) intravenous methylprednisolone given for 5 days in high-risk, severe (i.e. Kobayashi score ≥ 5) Japanese patients; when fever settled, this was then converted to oral prednisolone, tapered over 15 days after normalisation of CRP; versus a control group who received IVIG alone [18]. Again, the adverse event profile for both groups was comparable: serious adverse events occurred in 3/121 (2%) patients in the experimental group and 2/121 (2%) in the control group [18]. The types of serious adverse events were also similar between both groups: in the intravenous immunoglobulin plus prednisolone group, two patients had high total cholesterol, and one had neutropenia; and in the intravenous immunoglobulin group, one patient had high total cholesterol, and there was one episode of non-occlusive thrombus [18]. Importantly, observational data involving 724 high-risk Japanese patients (again identified by a high Kobayashi score) routinely treated with adjunctive corticosteroids also demonstrated minimal adverse events relating to corticosteroids, which occurred in only 2/724 patients (hypertension (*N* = 1); bacteraemia (*N* = 1)). Several more side effects were reported in relation to IVIG therapy, however [20]. This observational analysis therefore provides reassurance regarding the safety of corticosteroids in high-risk Japanese KD cases [20]. Lastly, meta-analysis of 16 comparative studies of 2746 patients with KD supported the use of corticosteroid therapy in severe KD (see above) and highlighted that efficacy was conferred without an increased risk of corticosteroid-related adverse events [21]. These data therefore suggest that overall corticosteroids are a safe treatment for KD.

#### Rationale for KD-CAAP

As summarised above, a number of recent studies conducted in different European countries (UK, Sweden and Germany), Russia and the United States have recently demonstrated alarmingly high rates of coronary complications despite IVIG [4, 8, 15–17]. These high complication rates now emphasise the need for an urgent reappraisal of IVIG and aspirin as the primary therapeutic agents for KD. Corticosteroids are an effective treatment for virtually all forms of vasculitis, but they have not been adopted as first-line treatment of unselected KD cases, for which there remains significant equipoise. Increasingly, evidence summarised above from randomised controlled trials and meta-analyses supports corticosteroid use as primary adjunctive treatment for patients with severe KD, particularly for Japanese patients with a Kobayashi score ≥ 5, and for patients regardless of ethnicity for whom CAA risk is 20–30% despite IVIG. This does not, however, resolve the ongoing debate about which KD patients should be considered as ‘severe’ outside of Japan, since the Kobayashi score and other clinical severity-scoring systems have poor predictive value in non-Japanese patients. Given however, the aforementioned high CAA complication rates seen across Europe (16–45%), all KD patients are arguably at high risk of CAA despite IVIG and could therefore potentially benefit from adjunctive corticosteroids as primary treatment for KD. Therefore, there remains significant equipoise regarding the use of corticosteroids as primary treatment combined with IVIG for all patients, i.e., not just the most severe cases. This protocol therefore describes a multi-centre randomised, controlled, open-label, blinded endpoint assessed, trial to explore the efficacy and safety of adjunctive corticosteroid therapy combined with IVIG/aspirin, versus IVIG/aspirin alone in unselected KD cases across Europe.

### Objectives {7}

The overarching goal of KD-CAAP is to optimise the treatment of KD in children/adolescents across Europe. KD-CAAP will test the hypothesis that adding immediate adjunctive corticosteroid treatment to IVIG and aspirin will reduce CAA rates in unselected KD patients across Europe compared with IVIG and aspirin alone.

The primary aim of the KD-CAAP trial is therefore to establish the effectiveness and efficacy of adjunctive corticosteroid therapy combined with IVIG and aspirin for prevention of CAA in unselected patients with KD across Europe.

Secondary aims are to establish:

1. The safety of adjunctive corticosteroid therapy combined with IVIG and aspirin for prevention of CAA in KD;
2. Whether adjunctive corticosteroid therapy reduces the duration of fever and length of hospitalisation for patients with KD;
3. The incremental cost-effectiveness ratio for corticosteroid therapy, expressed as the cost per QALY gained, from cost and utility data measured via resource use forms and the Child Health Utility 9D questionnaire.
4. The utility of the Paediatric Glucocorticoid Toxicity (pGTI) tool to assess corticosteroid toxicity [24].

### Trial design {8}

KD-CAAP is a multi-centre, randomised, open-label, blinded endpoint assessed, superiority trial of corticosteroids plus standard treatment with IVIG and aspirin, versus IVIG and aspirin alone for prevention of coronary artery aneurysms in KD.

## Methods: participants, interventions and outcomes

### Study setting {9}

KD-CAAP will be conducted in hospitals across several countries across Europe. The full list of study sites can be obtained at the following website: http://kdcaap.mrcctu.ucl.ac.uk/kd-caap-sites/.

### Eligibility criteria {10}

The inclusion criteria for KD-CAAP are:

1. Patients aged 30 days (post-natal age) to 15 years inclusive, and below the country-specific age of consent for the duration of the trial
2. KD defined in at least one of the three following ways:
  - As per the American Heart Association (AHA) criteria: namely fever for at least 5 days in addition to 4 of the following 5 clinical criteria [1]:
    (i) Bilateral non purulent conjunctivitis
    (ii) Cervical lymphadenopathy
    (iii) Polymorphous skin rash
    (iv) Changes in lips or mucosa (strawberry tongue, red cracked lips, diffuse erythematous oropharynx)
    (v) Extremity changes (erythema, oedema of palms and soles in initial phase, and at convalescent stage skin peeling)
  - OR less than 5 days of fever but all 5 clinical criteria above
  - OR incomplete KD cases, as per a modified AHA definition, namely:
    (i) Children/adolescents (> 1 year old) with fever greater than or equal to 5 days AND at least 2 other compatible clinical criteria as listed above; OR infants ≤ 1 year old with fever greater than or equal to 7 days without other explanation;
    AND for both age groups
    (ii) CRP ≥ 30 mg/L or erythrocyte sedimentation rate (ESR) ≥ 40 mm/h (or both)
    AND for both age groups
    (iii) EITHER the presence of any 3 or more of: anaemia for age (haemoglobin < lower limit of normal reference range for local laboratory) platelet count ≥ 450 × 10^9^/L or < 140 × 10^9^/L; albumin < 30 g/L; elevated ALT (> upper limit of normal reference range for local laboratory); white cell count ≥ 15 × 10^9^/L; urine ≥ 10 white blood cells per high power field
    (iv) OR abnormal echocardiogram compatible with KD but without established CAA, with ≥ 3 of the following suggestive features: decreased left ventricular function, mitral regurgitation, pericardial effusion, or dilated but non-aneurysmal coronary arteries (internal diameter 2 ≤ Z < 2.5; and not meeting the exclusion criteria for aneurysmal change as defined below).
3. Written informed consent from appropriate legal representative(s), and assent from patients who have not reached the age of consent and will not reach the age of consent for the duration of the trial in the participating country, but are judged to have capacity for this (depending on both age and acuity of illness)

This definition of incomplete KD is modified from the AHA definition (1) by firstly, the exclusion of aneurysmal coronary artery changes as the sole echo finding, since this is an exclusion criterion for KD-CAAP, and secondly the inclusion of low platelet count as well as high platelet count, as highlighted in recent European consensus SHARE guideline (2).

Disease-related exclusions include:

- This diagnosis is a second or further episode of KD.
- Already established CAA at screening.
- Severe congestive heart failure or cardiogenic shock defined as the presence of hypotension and shock requiring the initiation of volume expanders.
- Known congenital coronary artery abnormality that would impair assessment of the primary endpoint.
- Suspected macrophage activation syndrome.

Exclusions related to medications:

- Started IVIG more than 24 h prior to randomisation.
- Known hypersensitivity to prednisolone or methylprednisolone, or known phenylketonuria to aspartame used in a formulation in an infant less than 12 weeks.
- Current oral, intravenous, or intramuscular corticosteroid treatment for more than 3 days in previous 7 days prior to randomisation.
- History of previous severe reaction to any human immune globulin preparation.

Exclusions related to general health or other issues:

- Active varicella zoster virus or influenza infection; or known exposure to a case of varicella within the previous 21 days prior to randomisation if known to be non-immune.
- Co-enrolment in another study/trial of an investigative medicinal product.
- Pregnant or/and breastfeeding adolescents.

Disease-related exclusions relate to those (rare) patients who already have severe fulminant inflammation and/or shock when they are diagnosed with KD, in whom recent European consensus suggests corticosteroids and/or other immunosuppression are required. Such exceptional cases represent a small minority and therefore will not substantially impact on recruitment targets. A blood or urine pregnancy test must be completed on the day or day before randomisation for adolescents who have begun menstruation.

### Who will take informed consent? {26a}

Consent will be taken by a trial investigator during inpatient or outpatient emergency room visit. As children will be involved (aged 30 days to 15 years inclusive) consent will be obtained from parents or carers and assent from older children (dependent the acuity of illness and country requirements). Due to the acute nature of KD consent will be required promptly after KD diagnosis, a short trial introductory leaflet may initially be given to a potential patients, parent or carer after consideration and if requested the informed consent form will be given to the parent carer to provide consent. Consent for the use of the child’s medical, personal and samples for further research once the study is complete may be requested and the request to approach them in the future to find out how their child is doing. Also consent may be obtained before informing the patients’ local doctor of their participation in the trial.

### Additional consent provisions for collection and use of participant data and biological specimens {26b}

Consent will be also obtained for extraction and storage of DNA and RNA and stored plasma samples and serum.

## Interventions

### Explanation for the choice of comparators {6b}

The high CAA complication rates associated with KD seen across Europe (16–45%) suggest that all patients are arguably at high risk of CAA despite IVIG, and could therefore potentially benefit from additional primary treatment. Corticosteroids are an effective treatment for virtually all forms of vasculitis, but they have not been adopted as first-line treatment of unselected KD cases, for which there remains significant equipoise. KD-CAAP will therefore explore the efficacy and safety of adjunctive corticosteroid therapy combined with IVIG and aspirin compared to IVIG and aspirin alone (standard of care) in unselected KD cases across Europe.

### Intervention description {11a}

The investigational medicinal products within the trial are:

- IVIG (Human normal immunoglobulin)
- Aspirin
- Prednisolone or IV methylprednisolone

All children/adolescents will commence treatment with IVIG (2 g/kg which should be given as per local standard of care) and oral aspirin (40 mg/kg/day) as per standard of care. Children/adolescents will be randomised no more than 24 h after IVIG was initiated to two groups in a 1:1 ratio:

- Control group: no additional treatment
- Experimental group: adjunctive open-label oral prednisolone (2 mg/kg/day) or IV methylprednisolone equivalent (total 1.6 mg/kg/day) if oral prednisolone is not tolerated (for example, due to inability to take oral medication).

**Fig. 1:**
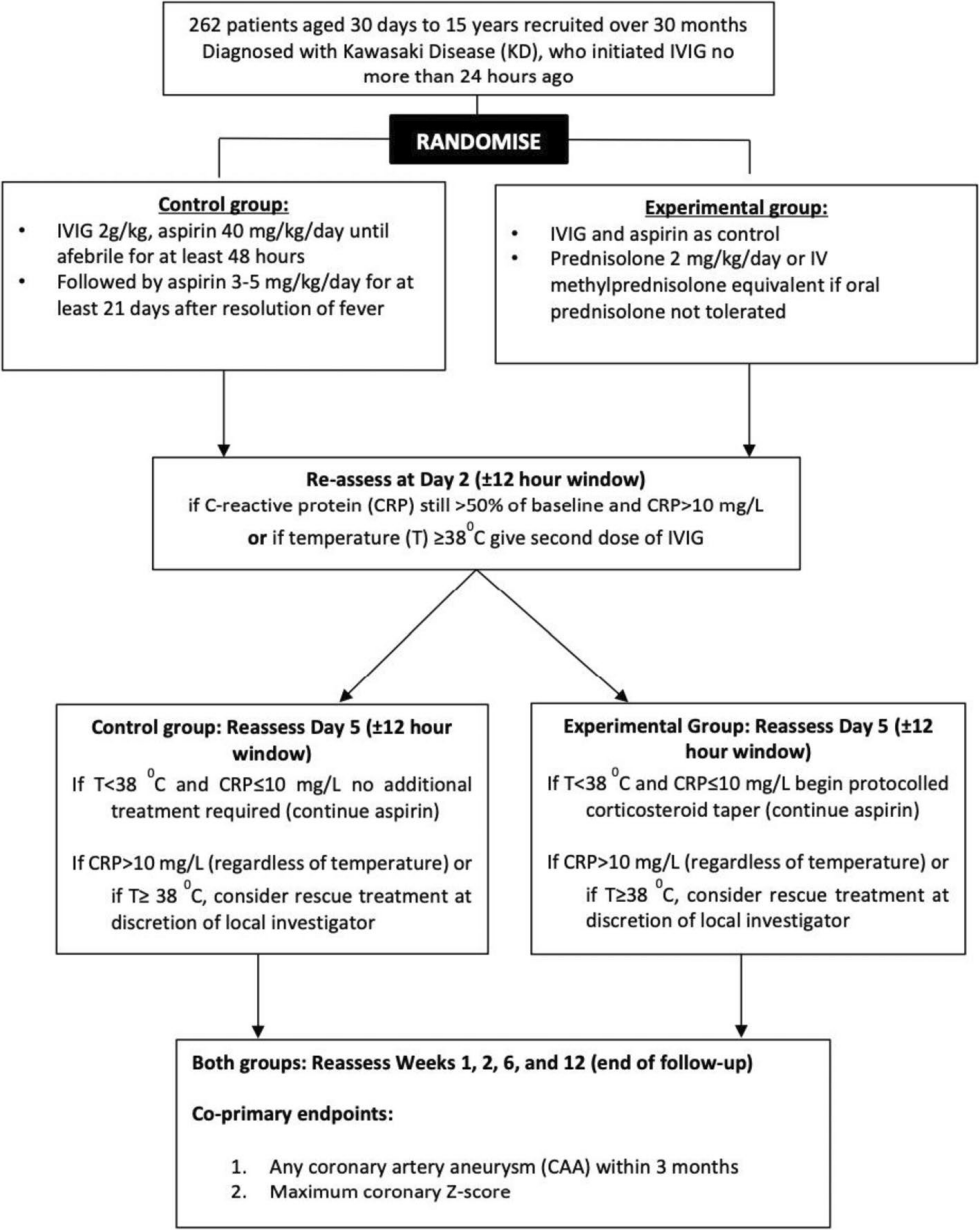
KD-CAAP trial schema

Further management will be dictated by temperature (body temperature) and C-reactive protein (CRP) responses (Fig. 1; and more details provided below).

#### Intravenous immunoglobulin and aspirin for both randomised groups

All children/adolescents will initiate treatment with IVIG (2 g/kg) and oral aspirin (40 mg/kg/day) as per standard of care. The local pharmacy stock of IVIG and aspirin will be used at each site. The particular formulation for IVIG will be that normally used at the local site, i.e. a particular preparation is not specified. The only specification is that this must be a preparation manufactured to appropriate medicinal product standards in Europe. This should be infused as per local standard of care, with intra-infusion monitoring as per standard of care for IVIG infusion at the local institution.

Aspirin will be administered orally, initially at 40 mg/kg/day in 4 divided doses until the child/adolescent is afebrile for at least 48 h; reducing to 3–5 mg/kg/day in one dose until at least 21 days after the resolution of the fever. Again, no particular preparation is specified; the site will use whichever preparation they normally use as per their routine clinical care. The oral formulation (tablet versus dispersible) will be determined by the age of the patient (usually dispersible in very young patients).

#### Control group: further management based on assessment of fever and CRP responses on day 2 and on day 5

Patients in the control group will be further assessed on follow-up day 2 (± 12 h). A second dose of IVIG (2 g/kg) can be given at this assessment if patient has CRP > 50% of baseline and still > 10 mg/L, or temperature (T) is ≥ 38 °C. Table 1 below summarises all the possible case scenarios based on CRP and temperature responses, and the treatment plan for each scenario.

**Table 1:** Management of patients in control group on day 2

Temperature	CRP	Treatment plan
< 38 °C	≤ 10 mg/L	No further treatment required [reassess on day 5]; reduce aspirin to 3–5 mg/kg/day when afebrile for at least 48 h and continue for at least 21 days after resolution of fever^a^
< 38 °C	> 10 mg/L but ≤ 50% of baseline	No further treatment required [reassess on day 5]; reduce aspirin to 3–5 mg/kg/day when afebrile for at least 48 h and continue for at least 21 days after resolution of fever^a^
< 38 °C	> 10 mg/L and still > 50% of baseline	Second dose of IVIG; reduce aspirin to 3–5 mg/kg/day when afebrile for at least 48 h and continue for at least 21 days after resolution of fever^a^
≥ 38 °C	≤ 10 mg/L	Second dose of IVIG; continue with aspirin at 40 mg/kg/day until afebrile
≥ 38 °C	> 10 mg/L but ≤ 50% of baseline	Second dose of IVIG; continue with aspirin at 40 mg/kg/day until afebrile
≥ 38 °C	> 10 mg/L and still > 50% of baseline	Second dose of IVIG; continue with aspirin at 40 mg/kg/day until afebrile

^a^ Following local standard of care

Flexibility in dose of ± 20% from the weight based dose is allowed for IVIG and aspirin.

At day 5 (± 12 h), further management is again dictated by temperature and CRP, as per below:

1. If CRP ≤ 10 mg/L and T < 38° C, no further additional treatment is required. Aspirin should be continued as per above.
2. If CRP > 10 mg/L or T ≥ 38° C rescue treatment should be considered at discretion of local investigator. See section below for recommended (but non-mandatory) rescue treatment options.

Table 2 below summarises the possible scenarios for management of patients in the control group based on assessment of temperature and CRP at day 5.

**Table 2:** Management of patients in control group at day 5

Temperature	CRP	Treatment plan
< 38 °C	≤ 10 mg/L	Continue aspirin at 3–5 mg/kg/day for at least 21 days after resolution of fever^a^; no further treatment required
< 38 °C	> 10 mg/L	Consider rescue treatment at discretion of local investigator; continue with aspirin at 3–5 mg/kg/day for at least 21 days after resolution of fever^a^
≥ 38 °C	≤ 10 mg/L	Consider rescue treatment at discretion of local investigator; continue with aspirin at 40 mg/kg/day until afebrile
≥ 38 °C	> 10 mg/L	Consider rescue treatment at discretion of local investigator continue with aspirin at 40 mg/kg/day until afebrile

^a^ Following local standard of care

#### Experimental group treatment

The local pharmacy stock of prednisolone/methylprednisolone will be dispensed for trial treatment as the trial IMP. The following licensed preparation of corticosteroids may be used:

- Methylprednisolone sodium succinate powder and solvent for solution of injection.
- Prednisolone tablets
- Prednisolone soluble tablets
- Prednisolone solution
- Prednisone tablets

These preparations are considered bioequivalent with appropriate dose adjustment (i.e. 1 mg prednisolone/prednisone equates to 0.8 mg of IV methylprednisolone) (https://bnf.nice.org.uk/treatment-summary/glucocorticoid-therapy.html). Prednisolone is a medicine with high solubility and high permeability (Biopharmaceutics Classification System—BCS class I). This means two immediate-released products (e.g. soluble tablet versus normal tablets) are likely to be bioequivalent (assuming similar excipients). A bioequivalence study was not considered necessary to support the licensing of prednisolone oral solution. It was considered bioequivalent to soluble tablet and to another prednisolone solution.

The experimental group will receive oral prednisolone at a dose of 2 mg/kg/day as soon as possible following randomisation. Maximum daily dose of oral prednisolone in first week (and subsequently) is 80 mg. If oral prednisolone is not tolerated then intravenous methylprednisolone may be given at equivalent doses (1.6 mg/kg/day, i.e. 0.8 mg/kg IV every 12 h). Corticosteroid tapering is allowed from day 5 onwards provided there is resolution of fever (temperature < 38 °C) and CRP ≤ 10 mg/L, and should be completed over 15 days in 5-day steps from 2 to 1 to 0.5 mg/kg/day, then to 0 mg.

Oral prednisone may be substituted for prednisolone at the same dose at the discretion of the local investigator following usual practice. Single daily dose soluble forms are acceptable in children.

Doses should be rounded to the nearest milligrams (easily achievable using soluble forms) that allow whole tablets to be administered (in accordance with dose ranges permitted). Flexibility in dose of ± 12.5% (but not exceeding 80 mg daily) from the milligram per kilogram dose above is allowed.

Proton pump inhibitor should be considered, e.g. lansoprazole 15–30 mg/day (or alternative proton pump inhibitor) until prednisolone dose is ≤ 10 mg/day (or 0.15 mg/kg/day), but are not mandated by the trial. Standard paediatric lansoprazole dosing will apply: for example children/adolescents < 30 kg 0.5–1 mg/kg (max dose of 15 mg) once daily; for children/adolescents ≥ 30 kg 30 mg once daily.

#### Experimental group: further management based on assessment of fever and CRP response on day 2 and day 5

As for the control group, a second dose of IVIG (2 g/kg) can be given on day 2 (± 12 h) if CRP > 50% of baseline and CRP > 10 mg/L, or if temperature ≥ 38 °C.

Table 3 below provides all possible case scenarios for management of the experimental group on day 2 IVIG.

**Table 3:** Management of patients in the experimental group on day 2 follow-up visit

Temperature	CRP	Treatment plan
< 38 °C	≤ 10 mg/L	Continue with prednisolone at 2 mg/kg/day no additional treatment required [reassess on day 5]; reduce aspirin to 3–5 mg/kg/day when afebrile for at least 48 h and continue for at least 21 days after resolution of fever^a^
< 38 °C	> 10 mg/L but ≤ 50% of baseline	Continue with prednisolone at 2 mg/kg/day no additional treatment required [reassess on day 5]; reduce aspirin to 3–5 mg/kg/day when afebrile for at least 48 h and continue for at least 21 days after resolution of fever^a^
< 38 °C	> 10 mg/L and still > 50% of baseline	Continue with prednisolone at 2 mg/kg/day and administer second dose of IVIG; reduce aspirin to 3–5 mg/kg/day when afebrile for at least 48 h and continue for at least 21 days after resolution of fever^a^
≥ 38 °C	≤ 10 mg/L	Continue with prednisolone at 2 mg/kg and administer second dose of IVIG; continue with aspirin at 40 mg/kg/day until afebrile
≥ 38 °C	≤ 10 mg/L but ≤ 50% of baseline	Continue with prednisolone at 2 mg/kg/day and administer second dose of IVIG; continue with aspirin at 40 mg/kg/day until afebrile
≥ 38 °C	≤ 10 mg/L and still > 50% of baseline	Continue with prednisolone at 2 mg/kg/day and administer second dose of IVIG; continue with aspirin at 40 mg/kg/day until afebrile

^a^ Following local standard of care

At day 5 (± 12 h) further management is dictated by temperature and CRP:

1. If CRP ≤ 10 mg/L and T < 38 °C, taper corticosteroids as described below. Aspirin should be continued as per above.
2. If CRP > 10 mg/L or T ≥ 38 °C rescue treatment should be considered. See below for suggested (non-mandatory) rescue treatments.

Table 4 below provides all possible case scenarios for management of patients in experimental group at day 5.

**Table 4:** Management of patients in the experimental group at day 5

Temperature	CRP	Treatment plan
< 38 °C	≤ 10 mg/L	Corticosteroid taper: oral prednisolone 1 mg/kg/day for 5 days, then 0·5 mg/kg/day for another 5 days, then stop; continue aspirin at 3–5 mg/kg/day for at least 21 days after resolution of fever^a^
< 38 °C	> 10 mg/L	Continue oral prednisolone 2 mg/kg/day until afebrile AND CRP ≤ 10 mg/L (then taper as above) and consider rescue treatment at discretion of local investigator; continue aspirin at 3–5 mg/kg/day for at least 21 days after resolution of fever^a^
≥ 38 °C	≤ 10 mg/L	Continue oral prednisolone 2 mg/kg/day until afebrile AND CRP ≤ 10 mg/L (then taper as above) and consider rescue treatment at discretion of local investigator; continue with aspirin at 40 mg/kg/day until afebrile
≥ 38 °C	> 10 mg/L	Continue oral prednisolone 2 mg/kg/day until afebrile AND CRP ≤ 10 mg/L (then taper as above) and consider rescue treatment at discretion of local investigator; continue with aspirin at 40 mg/kg/day until afebrile

^a^Following local standard of care

#### Rescue treatment

The trial schema (Fig. 1) and the tables above detail the management that children/adolescents in the trial should receive, including consideration of rescue treatment at day 5 based on fever and CRP responses. However, the local physician may add rescue treatment at any time if this is considered in the best interests of the child/adolescent; wherever possible, this should be discussed with the Chief Investigators first, and reasons for this will be recorded on CRFs. Rescue treatments will be chosen by the local investigator in line with their sites’ preferred standard of care. These non-mandatory rescue treatments may include:

(i) Re-treatment with IVIG (2 g/kg)
(ii) Corticosteroids: options (at the discretion of the local investigator) may include IV methylprednisolone at 10–30 mg/kg/day for 3 days followed by course of oral prednisolone at 2 mg/kg/day until there is resolution of fever and CRP≤ 10 mg/L; starting oral prednisolone (2 mg/kg/day) for 5 days if not previously received; continuation of oral prednisolone at 2 mg/kg/day for experimental group beyond day 5 until fever resolved and CRP≤ 10 mg/L
(iii) Infliximab 6 mg/kg/dose up to maximum of 2 doses 2 weeks apart
(iv) Ciclosporin at 5 mg/kg per day
(v) IL-1 blockade therapy (e.g. anakinra 2–4 mg/kg/day subcutaneously for 2 weeks, or longer depending on the therapeutic response)
(vi) Other therapies can also be considered at the discretion of the site investigator.

All children/adolescents receiving rescue treatments should continue to be followed up in the trial to the last 12-week follow-up visit, ‘on-study, off-study-treatment’, regardless of reason for initiating rescue treatment.

### Criteria for discontinuing or modifying allocated interventions {11b}

Adverse events caused by corticosteroid toxicity leading to a treatment change are expected to be rare. In the situation where this occurs, treatment may be discontinued at the discretion of the local investigator. Children/adolescents should remain in the trial for follow-up and should continue to follow the assessment schedule. Further treatment can be considered based on local investigator discretion. Lastly, if corticosteroids are stopped early, this should be tapered over several days (in accordance to local practice, i.e. not protocolised) to prevent adrenocortical insufficiency, in line with routine clinical care. The risk of gastrointestinal ulceration and bleeding may be increased when acetylsalicylic acid and corticosteroids are co-administered. This risk can be mitigated by the use of non-mandatory of proton pump inhibitors as per local practice.

We emphasize that an important goal of KD-CAAP is to document corticosteroid toxicity as well as effectiveness and efficacy. Although corticosteroids have been used for decades for the treatment of inflammatory diseases of the young, hitherto there has been no systematic method or tool to collate corticosteroid- related toxicity. We and others have recently developed such a tool for use in adults, the glucocorticoid toxicity index. We have now been involved in the development of a similar tool for use in paediatric trials: the paediatric Glucocorticoid Toxicity Index (pGTI) [24]. The pGTI consists of a Composite Index and a Specific List [24]. The Composite pGTI reflects glucocorticoid toxicity that has the potential to change during a clinical trial: to worsen if glucocorticoid doses increase, or to improve if successful glucocorticoid sparing is achieved. Toxicities included in the Composite pGTI are expected to occur commonly and to vary with glucocorticoid exposure [24]. They are therefore weighted and an aggregate score calculated. In contrast, the Specific List is designed to capture glucocorticoid toxicity not included in the Composite pGTI; these are often clinical events that are not reversible on lower corticosteroid exposure, or are uncommon (typically affecting ˂5%). The Composite Index of the pGTI consists of ten domains of glucocorticoid toxicity: body mass index, growth, glucose tolerance, lipid metabolism, systolic blood pressure, bone mineral density, glucocorticoid-induced myopathy, skin toxicity, neuropsychiatric impact and infections [24]. The Specific List includes six additional unique domains that address other features of glucocorticoid toxicity such as pubertal delay, sex hormone access interruption, ocular toxicity (cataracts, central serous retinopathy) and bone health (osteonecrosis) [24].

### Strategies to improve adherence to interventions {11c}

Corticosteroids will be initiated whilst the child is in hospital. In hospital, they will be administered by ward nurses and recorded on CRFs by trial staff. Therefore, non-adherence will be minimal. Intravenous methylprednisolone will only be given in hospital whilst the child is too unwell to tolerate oral medication. After discharge, we will document as accurately as possible what trial oral medication the patient actually takes, and the reasons for any reported noncompliance (including spitting out or refusing doses) using patient medication diaries for corticosteroids (as for aspirin). Corticosteroids are widely used worldwide for the treatment of a wide range of inflammatory diseases. The importance of adherence will be reinforced at the time trial medication is dispensed and during any subsequent contacts with the trial team. Formal assessment of corticosteroid-related toxicity, and how this may affect adherence, will also be assessed by using the pGTI [24].

### Relevant concomitant care permitted or prohibited during the trial {11d}

All regular medications will be recorded at enrolment. Parents will be asked to report the use of additional medications during follow-up visits. If a medication with a known major or moderate drug interaction with prednisolone or IVIG or aspirin is essential for a child’s management and cannot be replaced by a drug that does not have an interaction with these therapies, then the trial medication should be stopped and the concomitant medication used.

Since the main differential diagnosis for KD is infection, in line with routine clinical care, we anticipate that many children enrolled will be treated concomitantly with antibiotics (either oral or intravenous). Similarly, since KD is associated with fever, we anticipate that most, if not all, children will receive paracetamol (orally, as rectal suppository, or even intravenously if preferred by the standard of care at the recruiting site). Nonsteroidal anti-inflammatory drugs (NSAIDs) for the relief of pyrexia are contraindicated, however as discussed below. Antibiotics, paracetamol or any other medicines will be documented in the CRF.

In view of the age of participants eligible for inclusion in the trial (up to 15 years only), that pregnancy/breastfeeding is an exclusion criteria, the relatively short duration of follow-up (12 weeks), the fact that participants will be recruited when they are acutely unwell and hospitalised and will likely remain in hospital for at least a week and only be discharged on low-dose aspirin, and that corticosteroids and low-dose aspirin are commonly used in pregnancy the use of contraception is not expected in the trial.

Use of NSAIDs (e.g. ibuprofen, naproxen, indomethacin and mefenamic acid, amongst others) except aspirin is not allowed, because they abrogate the anti-platelet effect of low-dose aspirin therefore should be avoided following guidelines.

Immunisations should follow current recommendations regarding immunisations post IVIG. Immunisation with all live vaccines should generally be deferred for at least 6 months following an episode of KD treated with IVIG, mainly due to the potential lack of effectiveness following IVIG [1, 2]. Thereafter, all vaccines should be administered as recommended by national schedules. Currently available SARS-CoV-2 vaccines are not live and therefore guidance is as per any non-live vaccines.

### Provisions for post-trial care {30}

We do not anticipate that any of the patients recruited to KD-CAAP will need to continue any of the IMP beyond the 3 months duration of the trial. Post-trial care will be according to local practice and based on the discretion of the local treating clinical team.

The Sponsor of the trial is UCL. UCL holds insurance against claims from participants for injury caused by their participation in the clinical trial. Participants may be able to claim compensation if they can prove that UCL has been negligent. However, as this clinical trial is being carried out in a hospital, the hospital continues to have a duty of care to the participant of the clinical trial. This applies whether the hospital is a National Health Service Trust in the UK or otherwise. Participants may also be able to claim compensation for injury caused by participation in this clinical trial without the need to prove negligence on the part of UCL or another party. Participants who sustain injury and wish to make a claim for compensation should be advised to do so in writing in the first instance to the Chief Investigator, who will pass the claim to the Sponsor’s Insurers, via the Sponsor’s office. Institutions selected to participate in this clinical trial shall provide clinical negligence insurance cover for harm caused by their employees and a copy of the relevant insurance policy or summary shall be provided to UCL, upon request.

### Outcomes {12}

KD-CAAP will have two co-primary outcome measures:

1. Proportion of participants having any CAA (definition below) documented within the 12 weeks of trial follow-up (to assess overall effectiveness of the strategy of immediate corticosteroids in preventing CAA, expecting that some patients will receive rescue treatment before reaching this endpoint in both randomised groups).
2. An average estimate across weeks 1, 2 and 6 of the maximum of the *Z*-score of the internal diameters of the proximal right coronary artery or left anterior descending coronary artery, adjusting for rescue treatment (to assess the direct efficacy of corticosteroids).

CAA is defined as any of:

- Luminal diameter > 3.0 mm in a child < 5 years
- Luminal diameter > 4.0 mm in a child/adolescent ≥ 5 years
- Internal diameter of a segment at least 1.5 times that of an adjacent segment or when a
  - ◦ Luminal contour is clearly irregular
- Luminal internal diameter *Z*-score of ≥ 2.5. *Z*-scores for internal coronary artery diameter will be documented based on normative data: www.parameterz.com/refs/lopez-circimaging-2017.

CAA has been chosen as the primary endpoint because it is the most meaningful from the clinical viewpoint in terms of future risk of poor outcomes, and accords with previous trials in KD. CAA is defined as meeting the criteria at any time point—that is, it is a binary endpoint of the child ever experiencing this severe outcome. The intention-to-treat analysis of this endpoint therefore assesses the effectiveness of corticosteroids, i.e. is a real-world comparison of the intention to start corticosteroids as soon as possible vs not to start them immediately. To protect children, we have to allow children who are not doing well on their randomised therapy to switch to alternatives—it would not be ethical to propose maintaining children on randomised allocation until the point of developing CAA, which is what would be needed to consider the direct efficacy of corticosteroids vs no corticosteroids. This effectiveness comparison is arguably most relevant to clinicians, on the reasonable assumption that the kind of changes that occur to treatment during the trial would be similar to what would occur outside of the trial.

However, in order to estimate the direct efficacy of corticosteroids, we are proposing to conduct a novel inverse probability of (change from) treatment weighting co-primary analysis of the continuous outcome coronary diameter *Z*-score—we will have greatest power to identify differences between corticosteroids vs no corticosteroids (efficacy) with this continuous endpoint. The multiple values will be dealt with using standard repeated measures methods (most commonly generalised estimating equations with the weighting proposed to address efficacy).

The following secondary outcome measures will be assessed:

- Efficacy
-
  - At each of weeks 1, 2, 6 and 12 individually, the average maximum of the *Z*-score of the internal diameters of the proximal right coronary artery or left anterior descending coronary artery, adjusted for receipt of rescue treatment.
  - Proportion of participants having any CAA defined using a stricter definition of a luminal internal diameter Z-score of ≥ 2.5 alone documented within the 12 weeks of trial follow-up
  - Proportion receiving rescue treatment by week 12
  - Proportion receiving second dose of IVIG by week 12
  - Duration of fever after enrolment (time to temperature < 38 °C) assessed at 12 weeks.
  - Mean daily serum concentrations of CRP from days 1–5 and weeks 1–2, and averaged across these time points; and time to normalisation of CRP (defined as CRP ≤ 10 mg/L) assessed at 12 weeks.
  - Duration of hospitalisation (time to discharge) assessed at 12 weeks.

- Safety
-
  - Serious adverse events including deaths; number and proportion of participants having an event by week 12.
  - Grade 3 or 4 adverse events; number and proportion of participants having an event by week 12.
  - Clinical adverse events of any grade judged related to IVIG, aspirin or corticosteroids given to treat KD; number and proportion of participants having an event by week 12.

Other outcome measures that will be assessed are:

- Changes in other laboratory parameters of inflammation (haemoglobin, white cell count, platelet count, ESR, albumin); mean change from baseline estimated at day 2, day 5, week 2 and week 6 individually, and averaged across these time points.
- Duration of corticosteroid therapy; median (IQR) days within 12 weeks.
- Cumulative weight adjusted dose of prednisolone or methylprednisolone received; median (IQR) at week 12.
- Proportion of patients who need to continue prednisolone at 2 mg/kg/day beyond day 5
  - ◦ (experimental group, assessed at 12 weeks)
- Paediatric appropriate quality of life scores; mean change in total score from baseline to week 12.
- Paediatric corticosteroid toxicity index (pGTI) to assess glucocorticoid-related morbidity; mean change between week 1 and week 12.
- Incremental costs and cost-effectiveness (incorporating HRQL); budget impact (numeric tool specific readouts, median (IQR) at 12 weeks).

### Participant timeline {13}

The following table summarises the time schedule of screening, enrolment and assessments for participants.

Following randomisation, a window of 12 h either side of each trial visit will be permissible for visits up to D5, up to 1 day before and 3 days after for the week 1 assessment, up to 3 days either side of the week 2 assessment and up to 14 days either side for the week 6 and week 12 assessments. A visit on any given day from randomisation will only be counted once against the scheduled visit to which it is closest.

An unscheduled visit should be used to report a visit if it includes required assessment(s) that were not collected at the nearest scheduled visit, or to report any significant clinical event.

Every attempt should be made to keep the blood draw (including any losses in the manoeuver) for research samples within the 3% of the total blood volume recommended for children/adolescents during a period of 6 weeks for all children weighing over 8.75 kg and will not exceed 1% at any single time for all children weighing over 6.87 kg (since the total volume of blood is estimated at 80 to 90 ml/kg body weight, 3% equates to 2.4 ml blood per kg body weight). For any enrolled children lighter than 6.87 kg, the clinician should endeavour to ensure that the total blood volume for research samples does not exceed 1% at any single time. Since KD is an acute, severe illness, bloods required for routine clinical may occasionally surpass these limits as is often the case when managing critically ill children in routine clinical care.

### Sample size {14}

KD-CAAP adopts a novel methodological approach utilising two co-primary outcomes: one assessing the binary intervention of effectiveness of prednisolone (development of CAA or not); the other assessing intervention efficacy based on a continuous numeric outcome (average of the maximum of the *Z*-score of the diameter of the proximal right coronary artery or left anterior descending coronary artery, estimated across weeks 1, 2 and 6), improving the clinical relevance of the trial and using inverse probability weighting methods to adjust for non-compliance with the randomised intervention.

The advantage of the binary CAA primary endpoint is that it is clinically meaningful in terms of future mortality risk, and has been used in previous KD trials.

The sample size calculation was performed in Stata. Our estimated sample size of 262 children/adolescents provides 80% power to detect a reduction in CAA from 20% (based on existing survey data, 1–4) to 8% (based on reductions seen in ‘high-risk’ children, 20–23) (two-sided alpha = 0.05). We assume that this endpoint can be completely ascertained, given its severity and the severity of the condition (meaning 3-month follow-up is likely to be almost complete). Recruiting 262 children/adolescents is realistic over 30 months.

There is, however, a possibility that more children/adolescents in the control group will move to rescue therapy than in the experimental group, resulting in dilution of any efficacy signal in relation to the binary endpoint of presence or absence of CAA. We therefore include a co-primary outcome of average maximum *Z*-score across weeks 1, 2 and 6; analysed using generalised estimating equations (GEE) with inverse probability weighting to account for censoring of children/adolescents at the time they initiate rescue treatment (non-compliance with randomised strategy). This analysis would adjust for baseline *Z*-score using three strata: missing (to allow for the fact that some children/adolescents may not have a scan performed before randomisation), below median, above median. As well as accounting for differences in rescue treatment, this endpoint is continuous, so provides greater power. At any time point, 262 children/adolescents provides > 80% power to detect changes in the maximum coronary artery *Z*-score of 0.4 times the standard deviation (two-sided alpha = 0.05), assuming 13% children/adolescents may have missing values. There are no data to inform what effect estimate could be anticipated on continuous *Z*-scores, and therefore this effect size is pragmatic, based on the sample size for the binary CAA endpoint above.

The two endpoints will be considered separately, each with a nominal 0.05 level of significance, reflecting the fact that KD is a relatively small population in which it is important to generate randomised unbiased evidence and consider its totality. The overall type I error will depend on the correlation between the two effect estimates which is unknown (no data available to estimate this), but will be estimated using bootstrapping at the final analysis. The two endpoints are addressing different aspects (efficacy vs effectiveness) on different outcomes (highly clinically relevant binary endpoint with lower intrinsic power as binary vs potentially less relevant continuous endpoint with higher power as continuous). Therefore, it would be entirely possible to have significance on one and not the other and the clinical judgement is that corticosteroids should still be used immediately in all children.

Given the size of the trial, subgroup analyses are planned only by minimisation factors (excluding country), namely age (< 1 vs ≥ 1 year) and gender.

### Recruitment {15}

We have estimated recruitment rates in at least 30 centres across up to 15 countries in Europe over a 30-month recruitment period (at the time of writing, we have requested a 12-month extension due to pandemic delays). The MRC CTU at UCL is managing the trial and will be responsible for co-ordination of centres recruiting from the UK. The conect4children national hubs will be responsible for co-ordination of recruitment from non-UK sites to enable centres to open to recruitment in a timely fashion and to meet recruitment targets. The KD-CAAP trial design is similar to current standard of care, requiring a similar number of visits as per standard care. Our focus group of children and young people affected by KD showed a clear desire for an evidence base. Patients and parents told us at the focus group that they worry about cardiac outcomes, and therefore are motivated to attend for assessments that include echocardiography at follow-up. The compliance rate is therefore anticipated to be good, and thus loss to follow-up rates very low (less than 2–3%), particularly since follow-up is relatively short (3 months).

Reliable and up-to-date epidemiological data for KD from the UK suggest an incidence of 4.55 per 100,000 children under the age of 5 years per annum. In a recent British Paediatric Surveillance Unit (BPSU) study, we had 553 cases over 2 years [4]. A KD-CAAP feasibility study circulated through the C4C network has indicated preparedness across 17 countries, with multiple centres willing to recruit to the trial. Survey responses across Europe (including the C4C network, but also the PRINTO network) also indicated a similar case load to that suggested by the UK BPSU study. Thus, recruitment of 262 KD patients from at least 30 centres across Europe will be achievable over 30 months.

## Assignment of interventions: allocation

### Sequence generation {16a}

Randomisation will be performed using minimisation with a built in random element (stratified, see below) at the CTU using a computer algorithm concealed from the investigators/trial management staff, and accessed by either CTU staff or delegated site staff online. Epidemiological data suggest worse outcomes in terms of CAA for very young patients (age < 1 years) and male children/adolescents, who will therefore form two key subgroup analyses [1–4]. There remains significant equipoise about the use of corticosteroids in this young age group, however, and therefore it is essential to include these patients in KD-CAAP. In order to balance the groups for these two factors (age < 1 versus ≥ 1 year, sex), randomisation will be stratified for these two factors, as well as for recruiting country.

### Concealment mechanism {16b}

Randomisation will be performed online. To randomise a child/adolescent, the information contained on a completed randomisation CRF will be entered into the online trial database, accessible from the local clinical sites and CTU, which will automatically check for eligibility. Only children/adolescents with a completed and verified screening and randomisation CRF on the database will be able to be randomised. Allocation will be made after eligibility has been confirmed through the online database and will be concealed until the point of randomisation when only the randomisation for the current child/adolescent will be provided.

### Implementation {16c}

Delegated member(s) of staff at each site will be responsible for carrying out the randomisation process restricted using role-based access. The details of the child’s treatment allocation will be notified to clinical staff, and the allocation cross checked between those randomising and those managing the child/adolescent clinically. If the MRC CTU at UCL are to process the randomisation, the screening and randomisation CRF should be securely sent via electronic media to staff at the MRC CTU at UCL. At the MRC CTU at UCL, staff will verify eligibility and perform the randomisation using the online system. The details of the child’s treatment allocation will be notified to the trial team at the site by email or phone within 1 h of the receipt of the randomisation form (during UK normal working hours).

## Assignment of interventions: blinding

### Who will be blinded {17a}

The trial is open label so there will no blinding for participants or for care providers, or for statisticians. Only the echocardiographers reviewing centrally the echocardiogram-related endpoints will be blinded to treatment assignment.

### Procedure for unblinding if needed {17b}

As both the care providers and the trial participants are unblinded, no unblinding procedures are required for KD-CAAP**.**

## Data collection and management

### Plans for assessment and collection of outcomes {18a}

The frequency of follow-up visits and assessments are detailed in the Trial Assessment Schedule (Table 5). Trial assessments will be performed on D0, D1, D2, D3, D4 and D5 post-randomisation and at weeks 1, 2 and 6. Thereafter, the final trial assessment will be carried out at week 12 (last follow-up visit for each individual patient) after randomisation. A window of 12 h either side of each trial visit will be permissible for visits up to D5, up to 1 day before and 3 days after for the week 1 assessment, up to 3 days either side of the week 2 assessment and up to 14 days either side for the week 6 and week 12 assessments. However, if a child/adolescent attends late, all information required at the missed visit should still be collected, even if they attend outside the window. Further, given the age of the participants and the acuity of the illness, visits that do not happen within these windows will not be considered as protocol deviations. Most of these trial assessments coincide with standard clinical assessments, thus no extra visits are required for the trial out-with routine clinical care, with the exception of the 12-week visit. These assessments are as follows:

(i) At each visit, history and physical examination (with emphasis on clinical features of KD), vital signs (heart rate, blood pressure) and temperature/documentation of fever (T ≥ 38 °C), concomitant medication, adverse events, blood tests (see below) and resource utilisation. Height (or length in young children) will be assessed at D0, W1, W2, W6 and W12, and weight will be assessed at D0, W6 and W12. For children still febrile and in hospital on D5, maximum daily temperatures will be collected until discharge or afebrile for 2 calendar days whilst still hospitalised.
(ii) Urine dipstick for glycosuria, proteinuria, haematuria at baseline D0, D2, D5, weeks 1, 2, 6 and 12 (formal laboratory analysis may also be used). Microscopy of urine for white cell count is not mandated post screening.
(iii) Electrocardiogram (ECG) and echocardiography at weeks 1, 2, 6 and 12 (see section below on detailed echocardiography assessment).
(iv) Paediatric glucocorticoid toxicity index (pGTI) at weeks 1 and 12. This tool will assess glucocorticoid-related morbidity scored in different domains relating to body mass index, arterial hypertension, lipids, skin, neuropsychiatric symptoms, glucose tolerance and infection risk, amongst others (data will be collected for this tool on CRFs).
(v) Health economics assessments: resource utilisation at every visit, as above, and Health related quality of life (HRQL) at D0 and at least week 1 or 2 (depending on date of discharge) and weeks 6 and 12.
(vi) Quality of life (QoL) will be assessed at D0 and week 12.
(vii) Blood measurements (full blood count, ESR, CRP, biochemistry, liver function tests, glucose) will be performed as in Table 5 and results recorded in CRFs.
(viii) Blood will also be taken for storage for additional scientific studies as in Table 5. Adherence with aspirin for the control and experimental group and adherence to corticosteroids will be assessed using standardised diaries.

**Table 5:** Schedule of assessments

	Screening	D0*	D1	D2	D3	D4	D5	W1	W2	W6	W12	Unscheduled visit
Patient information sheet	X											
Assessment of eligibility criteria	X											
Informed consent (including for sample storage and genetic tests)	X											
Randomisation		X										
History & physical examination, vital signs [1]	X	X	X	X	X	X	X	X	X	X	X	X
Haematology [2]	[X]	X^b^	[X]	X	[X]	[X]	X	[X]	X	X	[X]	[X]
C-reactive protein	[X]	X^b^	X	X	X	X	X	X	X	X	[X]	[X]
Biochemistry [3]	[X]	X^b^	[X]	X	[X]	[X]	X	[X]	X	X	[X]	[X]
Urinalysis [4]	[X]a	X^b^	[X]	X			X	X	X	X	X	
ECG	[X]	[X]	[X]	[X]	[X]	[X]	[X]	X	X	X	X	[X]
Echocardiography [5]	[X]^a^	[X]	[X]	[X]	[X]	[X]	[X]	X	X	X	X	[X]
Concomitant drugs & trial treatment		X	X	X	X	X	X	X	X	X	X	
Health-related Quality of Life and resource utilisation [6]		X						X	X	X	X	
Quality of Life [7]		X									X	
pGTI								X			X	
Pregnancy test [8]	X										X	
**Research samples to be stored (scientific substudies):**												
- Plasma serum storage (0.5–1 ml EDTA and 0.5–1 ml serum)		X		X			X		X	X		
- EDTA blood for DNA storage (0.5–1 ml)		X										
- RNA pax gene tube for RNA extraction (2–2.5 ml)		X		X			X		X			
- Throat swab		X										
Maximum total blood draw for research samples (ml)		5.5	0	4.5	0	0	4.5	0	4.5	2	0	0

Randomisation occurs on day 0 (D0)

^a^Microscopy of urine for white cell count and echocardiography are only mandated at screening if required as inclusion criterion for incomplete KD

^b^Do not need to be repeated at randomisation if values are available on the day of randomisation or the day before randomisation from routine tests

[] indicates test not mandatory for the trial but results will be collected if available from routine care

[1] Including vital signs (temperature, heart rate, blood pressure (BP), adverse events. Height (or length in young children) will be assessed at D0, W1, W2, W6 and W12. For children still febrile and in hospital on D5 daily maximum temperatures will be collected until discharge or until afebrile for 2 calendar days to allow assessment of the secondary endpoint ‘duration of fever after enrolment’. Weight will be assessed at D0, W6 and W12

[2] Haematology: Hb, MCV, WCC, lymphocytes, neutrophils, platelets, erythrocyte sedimentation rate (ESR)

[3] Biochemistry: urea, creatinine, aspartate aminotransferase (AST), alanine aminotransferase (ALT), bilirubin, sodium, potassium, albumin, calcium, phosphate, glucose, alkaline phosphatase (ALP), lactate dehydrogenase (LDH)

[4] Urinalysis for proteinuria, haematuria, glycosuria

[5] Echocardiography baseline scan is not required for eligibility

[6] Child Health Utility 9D (CHU9D) questionnaire and EQ-5D-Y (youth version), depending on age, see Table 6 and if questionnaires are available in respective countries language. Should be completed at least one of W1 or W2 depending discharge. Resource utilisation will not be collected at baseline

[7] Paediatric quality of life (PedsQL™)

[8] Urine or blood pregnancy test must be completed for adolescents who have begun menstruation

#### Echocardiography and ECG

Two-dimensional echocardiograms will be digitally recorded at recruiting sites and interpreted at a core laboratory by at least one of two paediatric echocardiographers who will be blinded to randomised group. Echocardiography will be undertaken at weeks 1, 2, 6 and 12; any results of echocardiography done before enrolment or at any unscheduled timepoints will also be reviewed centrally. Echocardiography should include the following parameters:

- Assessment of cardiac function—normal/global dysfunction/regional dysfunction;
- Ejection fraction (biplane Simpson method);
- Left ventricular end diastolic diameter(LVEDD) and left ventricular end systolic diameter (LVESD) from M-mode;
- Assessment of mitral valve regurgitation—absent/mild/moderate/severe;
- Transmitral inflow characteristics including the peak early filling (Ewave) and late diastolic filling (A wave) velocities and the E/A ratio;
- Pulsed wave tissue Doppler Imaging (TDI) sampling from the septal and lateral mitral annulus including the early diastolic relaxation velocity (e′) and the systolic myocardial velocity (s’);
- Measurement of peak tricuspid regurgitation velocity;
- Measurement of diastolic left ventricular eccentricity index;
- Presence of pericardial effusion and depth in parasternal long axis plane;

In addition, coronary artery assessment and still frames of each coronary artery dimension with measurements should be obtained. Detailed measurements of internal diameters of the left main coronary artery (LMCA), left anterior descending (LAD) and right coronary artery (RCA) should be performed according to methodology described in Lopez et al. [25]. Height and weight must be measured accurately since these affect the body surface area for the *z*-scores. *Z*-scores for internal coronary artery diameter will be documented based on normative data: www.parameterz.com/refs/lopez-circimaging-2017. For the primary endpoint, CAA will be defined as luminal diameter > 3.0 mm in a child < 5 years; or > 4.0 mm in a child/adolescent ≥ 5 years; or internal diameter of a segment at least 1.5 times that of an adjacent segment or when a luminal contour is clearly irregular; or a luminal internal diameter *z*-score of ≥ 2.5. CAA defined by a luminal internal diameter *z*-score of ≥ 2.5 will be also considered as a standalone secondary endpoint. The type of coronary artery abnormality (saccular aneurysm, fusiform aneurysm, coronary ectasia) and the presence of thrombi (occlusive, non-occlusive) should also be recorded, as should the presence of pericardial effusion and valve regurgitation. Standard 12-lead ECG should be obtained at weeks 1, 2, 6 and 12, using standard equipment in clinical use at sites. There are no specific calibration requirements because this is not a formal endpoint, but an additional safety measure.

#### Health economics and quality of life measures

The health economic analysis will adopt the perspective of healthcare providers in each country. The health care costs for each patient will be estimated by collecting the use of healthcare resources, e.g. treatments, investigations, hospital admissions and contacts with health professionals on CRFs. Country-specific unit costs to value this resource use will be obtained from published and administrative sources. Outcomes in the economic analysis will be measured in terms of the co-primary outcomes of the trial, and in terms of health-related quality of life (HRQL) and quality-adjusted life years (QALYs) [26–28]. Health utilities suitable for measuring HRQL and QALYs will be assessed at day 0 and at least week 1 or 2 (depending on date of discharge) and weeks 6 and 12 using the Child Health Utility 9D (CHU9D) questionnaire to the parents/carers of all the participants and to the participants themselves if aged 8 years and over (Table 6). The CHU9D has been validated for use in children and is available in multiple languages. EQ-5D-Y (youth version) will additionally be administered to children/adolescents aged 8 years and over, and is available in multiple languages. The tools to be used according to the patients’ age are summarised in Table 6.

**Table 6:** Health economics questionnaires

Age	Completed by participant	Completed by parent / guardian (proxy)
< 1 to < 8 (from 30 days to 7 years inclusive))	None administered	CHU9D (proxy)
8 to < 16 (from age 8 to 15 years inclusive)	CHU9D EQ-5D-Y (including EQ-VAS -visual analogue scale)^a^	CHU9D (proxy)

^a^ EQ-5D-Y is recommended for 8–11 as well as 12–16 year olds (p4, https://euroqol.org/docs/EQ-5D-Y-User- Guide.pdf)

Quality of life (PedsQLTM score) is another endpoint that will be assessed within KD-CAAP [27, 28]. Relevant country-specific translations for these tools are available. It will be scored at day 0 and week 12. The PedsQLTM is a 23-item generic QoL questionnaire that has a child self-report for ages 5 through 18 years and a parent proxy report for children ages 2 through 18 years. The questionnaire takes 5 to 10 min to complete. The questionnaire yields information on the physical, emotional, social and school functioning of the child during the previous 4 weeks. It has been extensively tested in both healthy children and children with chronic disease. Mean scores are calculated based on a 5- point response scale for each item and transformed to a 0 to 100 scale with a higher score representing better quality of life. The PedsQLTM yields 3 summary scores: a total scale score, a physical health summary score and a psychosocial health summary score. There are 4 scale scores: physical functioning, emotional functioning, social functioning and school functioning. The total score is comprised of the average of all items in the questionnaire. The psychosocial summary score is comprised of the average of the items in the emotional, social and school functioning scales. The physical health summary score is comprised of the average of items in the physical functioning scale and is the same score as the physical functioning score.

### Plans to promote participant retention and complete follow-up {18b}

The expected lost to follow-up is likely to be minimal due to the duration of the trial. A parent/guardian who chooses to discontinue trial treatment for their child/adolescent should be encouraged to follow the trial procedures and follow-up schedule. However, if they do not wish to remain on trial follow-up, their decision must be respected and the child/adolescent will be withdrawn from the trial. The CTU should be informed of this in writing using the appropriate documentation. Prior to transferring to routine follow-up, the parent/guardian will be asked to have assessments performed as appropriate for a final trial visit. They would be at liberty to refuse any or all individual components of the follow-up assessments.

If follow-up is stopped early, the medical data collected during their participation in the trial will be kept and used in the analysis for the KD-CAAP trial, as consent cannot be withdrawn for data already collected. Similarly, samples and data obtained prior to this time will be processed according to the protocol for further research, unless the parent/guardian explicitly and unprompted requests otherwise. Consent for future use of stored samples already collected can be refused if follow-up is stopped early (but this should follow a discussion).

Given the short follow-up period (12 weeks), children/adolescents who have left the trial may not re-consent to participation in the trial subsequently.

Children/adolescents who stop trial follow-up early will not be replaced, since the sample size calculation already incorporates an inflation factor to account for lost-to-follow-up.

Procedures relating to ensuring queries are raised, managed and resolved appropriately are defined for the trial and includes methods of data cleaning and monitoring throughout the trial. The management of data quality and completeness is based on the trial risk assessment and managed through the trial oversight committees.

### Data management {19}

KD-CAAP will use an online database. Either site will be responsible for their own data entry directly.

onto the online trial database at the site or sites which can scan paper CRFs and email them to the MRC CTU at UCL for data entry. In both cases, the site will retain the original paper CRF. Data stored on the central database will be checked at MRC CTU at UCL for missing or unusual values (range checks) and checked for consistency within children/adolescents over time. If any problems relating to data quality are identified, the site will be contacted and asked to verify or correct the entry. Changes will be made on the original CRF and entered into the database at the site.

Paper CRFs, clinical notes and administrative documentation should be kept in a secure location (for example, locked filing cabinets in a room with restricted access) and held for a minimum of 25 years after the end of the trial. During this period, all data should be accessible, with suitable notice, to the competent or equivalent authorities, the Sponsor and other relevant parties in accordance with the applicable regulations. The data may be subject to an audit by the competent authorities. Medical files of children/adolescents in the trial should be retained in accordance with the maximum period of time permitted by the hospital, institution or private practice.

### Confidentiality {27}

The trial will be conducted in compliance with General Data Protection Regulation. In particular, the investigator must ensure that children’s anonymity will be maintained and that their identities are protected from unauthorised parties. Children/adolescents will be assigned a trial identification number and this will be used on CRFs; children/adolescents will not be identified by name. The investigator will keep securely a patient trial register showing trial numbers, name, date of admission and age at admission (in months or years). The unique trial number will identify all laboratory specimens, case record forms and other records and no names will be used, in order to maintain confidentiality. All records will be kept in locked locations. Clinical information will not be released without written permission, except as necessary for monitoring.

### Plans for collection, laboratory evaluation and storage of biological specimens for genetic or molecular analysis in this trial/future use {33}

The recruitment of large numbers of KD patients from multiple centres in Europe together with the collection of throat swab and low volume blood samples at multiple timepoints will be invaluable for future research on the diagnosis, aetiology, pathogenesis and genetics of the KD. So we propose a number of sub-studies in the context of KD-CAAP.

Over the past decade, there has been increasing evidence of the utility of RNA expression profiling to diagnose and understand the mechanisms involved in infectious and inflammatory diseases, and the development of minimal gene signatures that enable each condition to be diagnosed using small numbers of transcripts [29]. KD can be distinguished from a wide range of infectious and inflammatory disorders with as few as 13 gene transcripts. The small number of transcripts, and high level of sensitivity and specificity of the RNA signature, suggest that it could be developed as a diagnostic test. The KD-CAAP trial will enable validation of this approach, and investigation of whether RNA profiling can predict treatment response. Retrospective analysis of RNA samples will enable improved diagnostic accuracy of KD diagnosis for patients recruited to the trial. In parallel to RNA profiling, collection of serial serum and plasma samples will enable a parallel proteomic and metabolomic approach.

Collection of DNA, RNA, plasma and throat swabs from the trial cohort will advance research on mechanisms and aetiology of KD, through interrogation of the host response and of potential viral and bacterial pathogens or triggers. A major international effort is underway to identify precipitating infectious agents, including a metagenomic study of throat or nasopharyngeal aspirate samples. Samples taken on diagnosis from this trial will contribute to this larger study.

Genetic studies have shown important roles for gene variants in KD susceptibility and aneurysm formation. Patients recruited to the trial will be included in International KD Genetics studies**.** Protocols regarding collection, storage, analysis and eventual destruction of all biological materials are provided in the supplemental laboratory standard operating procedure (see supplemental material).

## Statistical methods

### Statistical methods for primary and secondary outcomes {20a}

The primary analysis population is intention-to-treat, ITT, which consists of all patients as randomised, regardless of treatment received (using inverse probability weighting to adjust for rescue treatment for the efficacy co-primary endpoint). Primary analysis will adjust for randomisation stratification factors, and secondary analyses will be unadjusted.

For the primary endpoints, analysis will include complete cases only if there is missing data in less than 10% of participants. If there is missing data in > 10%, for the binary endpoint multiple imputation (MI) by chained equations will be used to adjust for this. MI will be performed separately for each randomised group using logistic regression. If there is missing data, > 10% of participants for the continuous endpoint, additional probability weights will be used to adjust for this. All secondary endpoints will be analysed with complete cases only.

The CAA co-primary endpoint will be analysed using marginal effects after a logistic regression to calculate a risk difference and 95% confidence interval. The efficacy co-primary endpoint will be analysed using generalised estimating equations (GEE) with an independent correlation structure, with inverse probability weighting to account for censoring of children/adolescents at the time they initiate rescue treatment. This analysis will adjust for baseline *Z*-score using three categories: missing (as not all patients will have an echocardiogram at baseline), below median and above median. Weights will be calculated using logistic regression to determine the probability of receiving rescue treatment based on baseline characteristics (age, CRP and temperature at screening, baseline *Z*-score (if available, otherwise using a ‘missing’ group as above), country).

Analysis of secondary outcomes will use logrank tests and Cox regression for time-to-event outcomes, exact tests and binomial regression for binary outcomes, and *t*-tests and normal linear regression (potentially on log-transformed data depending on the observed data distribution, adjusted for baseline values) for continuous outcomes. Ranksum tests will be used if there is gross departure from normality that cannot be adequately addressed by data transformation. GEE with independent working correlation will be used to provide global tests of repeated measures. Adverse events will also be summarised by body system.

A Statistical Analysis Plan will be written and approved by the Trial Management Group (TMG), Trial Steering Committee (TSC) and the independent Data Monitoring Committee (DMC) before the first interim analysis is reviewed by the DMC.

For the economic analysis, we will multiply unit costs and resource use to generate total costs for every trial participant. We will do this using unit cost values for each country, allowing costs to be assessed from the health care provider perspective in each country. We will convert the HRQL measures into utility scores using country-specific tariffs where available and will use these to compute 3-month QALYs for every participant. We will then undertake a within-trial cost-effectiveness analysis calculating the incremental cost per unit of outcome gained, where outcomes will be measured separately in terms of the co-primary outcomes of the trial and QALYs. We will undertake extensive deterministic and probabilistic sensitivity analysis, including calculating cost-effectiveness acceptability curves. We will also use the incremental cost estimates combined with projected population sizes to calculate the budget impact of the new treatment regime.

### Interim analyses {21b}

The DMC will meet at least annually and will review trial data on recruitment, baseline characteristics, safety, adherence to randomised strategies and efficacy, as well as considering findings from any other relevant studies. The DMC may request additional analyses at any time as required for decisions on stopping, modifying or continuing the trial.

The DMC can recommend premature closure or reporting of the trial or that recruitment to any research group be discontinued or modified. Further details of DMC functioning, and the procedures for interim analysis and monitoring are provided in the DMC Charter.

The statistical stopping guideline for the trial is a Haybittle-Peto type rule based on the 99.9% confidence interval. At each review by the independent DMC, early stopping of the trial should be considered only if there is evidence beyond reasonable doubt (*p*-value < 0.001) of benefit on one or other of the co-primary endpoints. The independent DMC will also consider clinical criteria, other efficacy outcome(s) and safety outcomes in any decision about early stopping. Reasons will be recorded for disregarding a stopping guideline.

There are no stopping guidelines for futility because KD-CAAP is a pragmatic trial and all evidence regarding the potential benefits of corticosteroids adds to the evidence base, for example for future meta-analyses.

### Methods for additional analyses (e.g. subgroup analyses) {20b}

Given the size of the trial, subgroup analyses are planned only by minimisation factors (excluding country), namely age (< 1 vs ≥ 1 year) and gender. It is not known what percentages will fall into these different groups; depending on numbers (e.g. under 20% in one subgroup), these may not be possible. Subgroup analyses will be performed for the primary outcomes only and will be based on tests of interaction, although the range of the 95% CI will be used to identify the potential for greater harm or greater benefit in any subgroup. Any other subgroup analyses carried out will be considered exploratory and interpreted with extra caution. Subgroup analyses will be performed at the final analyses only.

### Methods in analysis to handle protocol non-adherence and any statistical methods to handle missing data {20c}

Analysis will be performed on an intention-to-treat basis including all randomised patients, regardless of the treatment received, making the generalizability assumption that the types of changes to treatment that happen in the trial are similar to those that would happen outside of the trial in this acutely sick population.

For the co-primary outcomes, if missing data or losses to follow-up are less than 10% of participants then analysis of primary outcomes will use observed data only. If there is missing data in > 10%, for the binary endpoint multiple imputation (MI) by chained equations will be used to adjust for this. MI will be performed separately for each randomised group using logistic regression. The imputation model will include age, sex, CRP and temperature at screening, baseline *z*-score (if available) and country. The imputation model includes the key covariates which are hypothesised to be most strongly associated with the outcome.

If there is missing data for the continuous endpoint in > 10% of participants, additional probability weights will be used to adjust for this. The missingness weights will be estimated using the same predictors as above in an initial approach; as the mechanisms underlying missingness are completely unknown, data exploration will be conducted to identify whether there are other important predictors. Weights for missingness and receipt of rescue treatment will be multiplied together, as in causal analysis approaches such as marginal structural models.

### Plans to give access to the full protocol, participant-level data and statistical code {31c}

The MRC CTU at UCL supports a controlled access approach to data sharing participant-level data and statistical code [30]. The protocol is publicly available on the MRC CTU website [31]. The data derived from this clinical trial are considered the property of the KD-CAAP TSC. Data will be shared according to the CTU’s controlled access approach [32]. Data will be available for sharing after publication of the primary trial results. Researchers wishing to access data should contact the Trial Management Group in the first instance.

## Oversight and monitoring

### Composition of the coordinating centre and trial steering committee {5d}

A Trial Management Team (TMT) will be formed to conduct the day-to-day management of the trial at the CTU. This will include the Co-Chief Investigators, trial statisticians, trial physician, clinical project manager, trial managers (TM) and data manager. The group will meet at least once per month, although may meet more often if required. The group will discuss issues related to the progress of the trial at the site and to ensure that the trial is running well. A Trial Management Group (TMG) will be formed comprising the two co-Chief Investigators, co- investigators and clinical and non-clinical members of the CTU. It will meet every 3–6 months depending on the stage of the trial generally by teleconference. This group will be chaired by the Chief Investigator(s) and all decisions regarding the overall running of the trial will be made in this forum with the exception of matters of fundamental importance to the viability of the trial or that require major changes to the protocol. These will be referred to the Trial Steering Committee (TSC). The Trial Steering Committee (TSC) has membership from the TMG plus independent members, including the Chair and community representatives. The role of the TSC is to provide overall supervision for the trial and provide advice through its independent Chair. The ultimate decision for the continuation of the trial lies with the TSC. Further details of TSC functioning are presented in the TSC Charter.

### Composition of the data monitoring committee, its role and reporting structure {21a}

An independent Data Monitoring Committee (DMC) will be formed. The DMC will be the only group who sees the confidential, accumulating data for the trial. Reports to the DMC will be produced by the CTU statisticians. The DMC is planned to meet within 12 months of the first participant recruited; the frequency of meetings will be determined by the DMC. The DMC will consider data using the statistical analysis plan (see Sect. 9.5) and will advise the TSC. The DMC can recommend premature closure or reporting of the trial or that recruitment to any randomised group be discontinued.

### Adverse event reporting and harms {22}

The definitions of the EU Directive 2001/20/EC Article 2 based on the principles of GCP apply to this trial protocol. These definitions are given in Table 7.

**Table 7:** Adverse events definitions

Term	Definition
Adverse event (AE)	Any untoward medical occurrence in a patient or clinical trial subject to whom a medicinal product has been administered, including occurrences that are not necessarily caused by or related to that product
Adverse reaction (AR)	Any untoward and unintended response to an investigational medicinal product related to any dose administered
Unexpected adverse reaction (UAR)	An adverse reaction, the nature or severity of which is not consistent with the information about the medicinal product in question, as set out in the Summary of Product Characteristics (SPC) or Investigator Brochure (IB) for that product
Serious adverse event (SAE) or serious adverse reaction (SAR) or suspected unexpected serious adverse reaction (SUSAR)	Any adverse event, adverse reaction or unexpected adverse reaction that: Results in death Is life-threatening^a^ Requires hospitalisation or prolongation of existing hospitalisation^b^ Results in persistent or significant disability or incapacity Consists of a congenital anomaly or birth defect Is another important medical condition^c^

^a^ The term life-threatening in the definition of a serious event refers to an event in which the patient is at risk of death at the time of the event; it does not refer to an event that hypothetically might cause death if it were more severe, for example, a silent myocardial infarction

^b^ Hospitalisation is defined as an inpatient admission, regardless of length of stay, even if the hospitalisation is a precautionary measure for continued observation

^c^ Medical judgement should be exercised in deciding whether an AE or AR is serious in other situations. The following should also be considered serious: important AEs or ARs that are not immediately life-threatening or do not result in death or hospitalisation but may jeopardise the subject or may require intervention to prevent one of the other outcomes listed in the definition above; for example, a secondary malignancy, an allergic bronchospasm requiring intensive emergency treatment, seizures or blood dyscrasias that do not result in hospitalisation or development of drug dependency

Adverse events include:

1. An exacerbation of a pre-existing illness
2. An increase in frequency or intensity of a pre-existing episodic event or condition
3. A condition (even though it may have been present prior to the start of the trial) detected after trial drug administration
4. Continuous persistent disease or a symptom present at baseline that worsens following administration of the trial treatment

Adverse events do not include:

- Medical or surgical procedures; the condition that leads to the procedure is the adverse event
- Pre-existing disease or a condition present before treatment that does not worsen
- Hospitalisations where no untoward or unintended response has occurred, e.g. elective cosmetic surgery
- Overdose of medication without signs or symptoms

Corticosteroids are commonly used in pregnancy (for example, for the treatment of recurrent miscarriage or foetal abnormalities such as congenital adrenal hyperplasia), and their benefits are judged to outweigh any risk. Similarly, IVIG and low-dose aspirin (to reduce pre-eclampsia) are used in pregnancy. Given the age range that will be recruited and short duration of follow-up, a pregnancy test for adolescents menstruating will be carried out at week 12. Adolescents who become pregnant within their trial participation will be followed up to pregnancy outcome. Administration of the trial IMP if pregnancy is identified should be managed by the local investigator taking into account the risks and benefits to both the participant, given the serious and acute nature of Kawasaki disease, and to the unborn child.

All AEs should be recorded in the patient’s medical notes. All grade 3 or 4 adverse events should be reported on the relevant CRFs, as should be adverse events of any grade that lead to modification of IVIG, aspirin or corticosteroids, clinical adverse events judged definitely/probably/possibly related to IVIG, aspirin or corticosteroids and any SAEs. SAEs should be notified to the CTU within 24 h of the investigator becoming aware of the event. When an AE or AR occurs, the investigator responsible for the care of the child/adolescent must first assess whether or not the event is serious using the definition given in Table 7. If the event is serious, then an SAE form must be completed and the CTU notified within 24 h.

The severity of all AEs and/or ARs (serious and non-serious) in this trial should be graded using the toxicity grading in The Division of AIDS (DAIDS) Table for Grading the Severity of Adult and Pediatric Adverse Events.

The investigator will assess the causality of all serious events or reactions in relation to the trial IMP using the definitions in Table 8, regardless of when during follow-up the event occurred. There are five categories: unrelated, unlikely, possible, probable and definitely related to receipt of the trial drug. If the causality assessment is unrelated or unlikely to be related, the event is classified as an unrelated SAE. If the causality is assessed as possible, probable or definitely related, the event is classified as an SAR.

**Table 8:** Assigning type of SAE through causality

Relationship	Description	SAE type
Definitely	There is clear evidence to suggest a causal relationship and other possible contributing factors can be ruled out	SAR
Probable	There is evidence to suggest a causal relationship and the influence of other factors is unlikely	SAR
Possible	There is some evidence to suggest a causal relationship (for example, because the event occurs within a reasonable time after administration of the trial medication). However, the influence of other factors may have contributed to the event (for example, the child’s clinical condition, other concomitant treatments)	SAR
Unlikely	There is little evidence to suggest that there is a causal relationship (for example, the event did not occur within a reasonable time after administration of the trial medication). There is another reasonable explanation for the event (for example, the child’s clinical condition, other concomitant treatment)	Unrelated SAE
Unrelated	There is no evidence of any causal relationship	Unrelated SAE

The CTU should be notified of all SAEs within 24 h of the investigator becoming aware of the event.

The minimum criteria required for reporting an SAE are the trial number, name of investigator reporting the event and why it is considered serious.

Medically qualified staff at the CTU and/or the Chief Investigator (or a medically qualified delegate) will review all SAE reports received. Events will be MedRA coded. The causality assessment given by the local investigator at the hospital cannot be overruled; in the case of disagreement, both opinions will be provided in any subsequent reports.

If the IMP’s causal relationship to the serious adverse event has been assessed as possible, probable or definitely, the CTU has the responsibility to determine the expectedness of the event to the trial IMP. An unexpected adverse reaction is one that is not listed within the trial Reference Safety Information or one that is more frequent or more severe than previously reported. If a SAR to the trial IMP is assessed as being unexpected, it becomes a SUSAR. Section 4.8 of a representative SPC will be considered as the Reference Safety Information. The trial Safety Management Plan will define the choice of SPC.

The CTU is undertaking the duties of the trial Sponsor and is responsible for the reporting of SUSARs and other SARs to the regulatory authorities (MHRA and competent authorities of other European member states and any other countries in which the trial is taking place) and the research ethics committees, as appropriate. Fatal and life-threatening SUSARs must be reported to the competent authorities within 7 days of the CTU becoming aware of the event; other SUSARs must be reported within 15 days. This responsibility may be delegated to one representative in each country for relevant reporting requirements in individual countries.

The CTU will also keep all investigators informed of any safety issues that arise during the course of the trial.

The CTU, as Sponsor, will submit once a year an Annual Safety Reports in the form of a Developmental Safety Update Report (DSUR) to Competent Authorities (Regulatory Authority and Ethics Committee). The DSUR will include:

1. A line list of all suspected (unexpected or expected) serious adverse reactions, along with an cumulative summary table of all reported serious adverse events, ordered by body system
2. A report concerning the safety of the subjects, consisting of a complete safety analysis and an evaluation of the balance between the benefit and risk of the IMPs under investigation.

### Frequency and plans for auditing trial conduct {23}

Authorised representatives of the sponsor and competent authority may conduct independent audits/inspections according to a pre-determined audit plan. Monitoring and source data verification will be conducted according to the quality and management monitoring plan.

### Plans for communicating important protocol amendments to relevant parties (e.g. trial participants, ethical committees) {25}

The MRC CTU at UCL will prepare the documentation for important protocol amendments including any documents that will need to be provided to participants involved in the trial (if required). These will be disseminated to the conect4children national hubs, who will be responsible for the translation and submission of the amendment to the ethical committees and competent authorities locally for the non-UK sites. For the UK sites, the MRC CTU at UCL will submit the amendment to the research ethics committee and Medicines and Healthcare products Regulatory Agency.

### Dissemination plans {31a}

The KD-CAAP TSC is the responsible for the data and specimens generated from the KD-CAAP trial; KD-CAAP trial data are not the property of individual participating investigators or health care facilities where the data were generated. It is anticipated that several opportunities will arise for publication during the course of and following completion of the KD-CAAP trial. Publications include papers (including abstracts) for presentation at national and international meetings, as well as the preparation of manuscripts for peer-reviewed publication. All publications are to be approved by the TMG and TSC before submission for publication. Any publication arising before the end of the trial (not by randomised groups) will also be approved by the DMC to ensure that the primary objective of the trial (the randomised comparison) is not compromised. No analyses by randomised group of any outcome (primary, secondary or other) in either the main trial or associated sub-studies will be conducted or presented before the end of the trial, other than those for interim review by the DMC. In line with MRC policy that the results of publicly funded research should be freely available, manuscripts arising from the trial will, wherever possible, be submitted to peer-reviewed journals which enable Open Access via UK PubMed Central (PMC) within 6 months of the official date of final publication. Wider public dissemination also falls into the remit of the patient engagement work which is being undertaken as part of KDCAAP facilitated by Societi UK.

## Discussion

Several recent studies have indicated that coronary complications associated with KD across Europe are much higher than early trials of IVIG had initially suggested [4, 8, 15–17, 33]. KD-CAAP directly addresses this issue by exploring the therapeutic benefit of adjunctive corticosteroids in unselected KD cases. If we find that corticosteroids prevent CAA, and are safe, this is a cheap and widely available intervention that would be implemented immediately for the benefit of children across Europe and likely North America where these issues are of similar concern.

One of the novelties of KD-CAAP is that it will have two co-primary end points and will explore both the effectiveness and efficacy of adjunctive corticosteroids in unselected KD cases, in contrast to previous studies which focused on predominantly severe Japanese KD cases. Despite several comparative and non-comparative studies comparing the impact of corticosteroids in KD, this potentially highly effective treatment is not commonly used across Europe [2]. Several factors likely explain this: inability to identify high-risk children early on in the disease course, when the meta-analysis suggests benefits will be greatest; lack of clarity on wider benefits in terms of longer-term cardiovascular health in children without overt vasculitis (coronary artery aneurysms, CAA); relative weakness of the evidence base with randomised trial evidence being relatively small; and concerns about generalizability of findings from Japanese studies to non-Japanese populations in case ethnic differences contribute to variable efficacy. KD-CAAP will delineate the evidence supporting adjunctive corticosteroids (or not), in all-comers with KD, leading to a pragmatic and easily implementable recommendation.

Historically, resolution of fever has been regarded as the sole metric of therapeutic success when treating acute KD [18, 19], but this is inadequate in isolation and may lead to false reassurance of therapeutic response. KD-CAAP includes check points for additional /rescue treatments to be added depending upon resolution of fever, and for the first-time normalisation of CRP. We have designed a trial that also actively manages the control group with rigorous assessments based on clinical status, fever and CRP to determine IVIG treatment failure in the control group, whilst keeping them corticosteroid-free. Such an approach was strongly supported by patient representatives and makes the trial ethically much more feasible. We highlight that KD-CAAP has been designed from its earliest inception with strong patient involvement (Societi UK). This close partnership with patient representatives will ensure successful delivery of the trial and dissemination of results to other patients and the wider public. Lastly, another novelty of KD-CAAP is the use of the pGTI to assess systematically corticosteroid toxicity in addition to adverse event reporting [24].

KD-CAAP is one of the academic proof of viability studies supported by the conect4children (c4c) network, a novel European research network that aims to facilitate drug research in children (https://conect4children.org). The specific goal of the c4c consortium is to set up and evaluate selected elements of a paediatric-focused clinical trial infrastructure that are tailored to meet the needs of children involved in clinical trials. The elements are as follows: expert advice providing input on study design and/or paediatric development programmes (including patient involvement activities); a network of sites following harmonised procedures and coordinated by national hubs (NH) and a single point of contact (SPoC) for Europe; a facility for education and training for sites and trial teams; and support for managing data used by the network and a common paediatric data dictionary. The work of c4c on trials is being evaluated through studies such as KD-CAAP that will test the viability of the network and will provide outputs such as reports on operational feasibility at country and site level, consistent support for trial conduct, and facilitation of timely opening and completion of trials.

We highlight that the risks of subjects’ involvement in KD-CAAP were also specifically assessed in the context of the global COVID-19 pandemic and the applicable precautionary response measures in place at both a local or national level for participating sites and countries. Together with c4c, we have worked on mitigating the impact of the COVID‐19 pandemic through a comprehensive business continuity plan. The main barriers to the progression of KD-CAAP were the availability of staff to work on the clinical studies or their ability to process contracts and submit applications for ethical and other regulatory approvals, and the prioritisation of COVID-related trials and studies over any other type of trial or research activity across several European countries. This has inevitably led to a delay of at least 12 months in site set-up, with a direct impact on recruitment timelines for KD-CAAP. The possibility of remote patient visits was also explored but this was not possible for the primary outcome assessment (echocardiography) and some other study procedures (blood tests). We have also explored remote monitoring, but some national regulatory authorities limit this approach. Shortage of supply of IVIG is currently closely monitored and so far has not impacted our progress.

An additional challenge we face relates to the diagnostic equipoise between KD and a novel hyperinflammatory syndrome in children associated with SARS-CoV-2 that has recently emerged, referred to as paediatric inflammatory multisystem syndrome temporally related to SARS-CoV-2 (PIMS-TS) [34]. PIMS-TS possibly represents a rare, delayed immune-mediated response rather than direct viral sepsis in a small minority of children [34]. Patients with PIMS-TS may have KD-like feature, but typically have shock requiring intensive care unit admissions [34]. KD-CAAP already excludes patients with cardiogenic shock and therefore so far has not been impeded by inclusion of patients with PIMS-TS. In addition, treatment of PIMS-TS and KD is essentially the same, with corticosteroids and IVIG forming the mainstay, further reducing risk of any harm to patients if the diagnosis changes after recruitment to KD-CAAP. Lastly, SARS-CoV-2 may trigger KD but this should not have any impact on treatment as patients should still receive KD treatment and be considered for recruitment to KD-CAAP irrespective of SARS-CoV-2 or any other infectious trigger. Lastly, we are currently considering some additional biomarker studies to examine the impact of SARS-CoV-2 PCR positivity and serology status on the results of KD-CAAP.

In summary, KD-CAAP is a multicentre randomised, controlled, open-label, blinded endpoint assessed, trial that for the first time explores the efficacy of adjunctive corticosteroids in the prevention of CAA in unselected cases of KD across Europe and is likely to lead to easily implementable changes in the management of KD.

## Trial status

We are currently working on protocol version 5, date 7th April 2021. Recruitment began on 2nd January 2021 and is scheduled to finish January 2024.

## Data Availability

The KD-CAAP TSC is the custodian for the data and specimens generated from the KD-CAAP trial; KD-CAAP trial data are not the property of individual participating investigators or health care facilities where the data were generated.

## Abbreviations

AE: Adverse event
AHA: American Heart Association
AR: Adverse reaction
C4C: Conect4children
CAA: Coronary artery aneurysm
CHU9D: Child Health Utility 9D
CI: Confidence interval
CRF: Case report form
CRP: C reactive protein
DMC: Data Monitoring Committee
DSMB: Data Safety Monitoring Board
HRQL: Health-related quality of life
IVIG: Intravenous immunoglobulin
KD: Kawasaki disease
KDCAAP: Kawasaki Disease Coronary Artery Aneurysm Prevention trial
MRC: Medical Research Council
MRC CTU: Medical Research Council Clinical Trials Unit at University College London (also generally abbreviated to ‘CTU’)
pGTI: Paediatric Glucocorticoid toxicity index
PIMS-TS: Paediatric Multisystem Inflammatory Syndrome
QALY: Quality-adjusted life year
RCT: Randomised controlled trial
REC: Research Ethics Committee
SAE: Serious adverse event
SAP: Statistical Analysis Plan
SAR: Serious adverse reaction
SHARE: Single Hub Access Point for paediatric Rheumatology in Europe
SOP: Standard operating procedure
SPC: Summary of Product Characteristics
SUSAR: Suspected unexpected serious adverse reaction
T: Temperature
TM: Trial Manager
TMG: Trial Management Group
TMT: Trial Management Team
TSC: Trial Steering Committee
UAR: Unexpected adverse reaction
UCL: University College London
